# Peri-articular diseases of the hip: emerging frontiers in arthroscopic and endoscopic treatments

**DOI:** 10.1007/s10195-013-0253-z

**Published:** 2013-07-28

**Authors:** A. Aprato, N. Jayasekera, A. Bajwa, R. N. Villar

**Affiliations:** 1 The Richard Villar Practice, Spire Cambridge Lea Hospital, Impington, Cambridge, CB24 9EL UK; 2 Corso Lanza 72, 10131 Turin, Italy

**Keywords:** Peri-articular hip diseases, Hip arthroscopy, Hip endoscopy

## Abstract

The precise diagnosis of both intra and extra-capsular disease of the hip is now possible because of commonly available advanced diagnostic imaging techniques. An increasing number of reports in the orthopedic literature describe new endoscopic and arthroscopic techniques to address peri-articular pathology of the hip. The purpose of this paper is to review current techniques in the management of extra-articular hip conditions.

## Background

Hip arthroscopy has gained considerable popularity in the past decade because of an increasing understanding of femoroacetabular impingement (FAI) [1]. At present FAI is the most common indication for hip arthroscopy, though several other intra-articular conditions of the hip such as loose bodies, labral tears and chondral lesion are also commonly treated [2]. Recent orthopedic literature has focused mainly on these hip conditions, but advances in diagnostic imaging techniques now allow for the precise diagnosis of extra-capsular disease of the hip, often with associated intra-capsular pathology. Furthermore, an increasing number of surgeons have recently described arthroscopic and endoscopic techniques in addressing peri-articular conditions of the hip [3].

The purpose of this paper is to review current techniques in the management of extra-articular hip conditions.

**Fig. 1:**
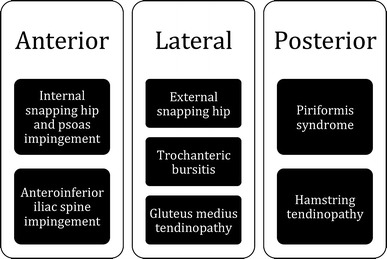
Anatomic classification

For academic purposes we divide peri-articular hip diseases according to the anatomical compartment involved (Fig. 1). In the following paragraphs we describe each pathological anatomic structure and appropriate arthroscopic or endoscopic treatment.

### Internal snapping hip syndrome and psoas tendon impingement

The internal snapping hip syndrome may be caused by the iliopsoas tendon [4]. The snapping usually results as the hip is extended from a flexed position, usually from more than 90º of flexion. In this position the iliopsoas tendon may snap over the iliopectineal eminence or the femoral head.

Other causes of snapping should be excluded: the snap could be produced by the iliotibial band snapping over the greater trochanter, due to intra-articular pathology, such as loose bodies or labral tears [4]. This is no longer referred to as a snapping hip because the diagnosis of intra-articular pathology is now more accurate with MRA [5].

Symptoms and clinical examination of external snapping syndrome will be described in the following paragraphs, and ultrasound examination can verify the snap between greater trochanter and iliopsoas.

Although the internal snapping phenomenon can’t be demonstrated directly with magnetic resonance arthrography (MRA), magnetic resonance imaging may frequently report changes within the iliopsoas tendon and bursa [5].

Asymptomatic internal snapping of the hip may occur in up to 10 % of the general population. It is considered within normal variance and is thus managed conservatively [4]. The symptomatic internal snapping hip syndrome defined by pain in the groin is, however, a separate matter. Physiotherapy is the first line of treatment even in this scenario [4], with arthroscopic treatment reserved for those in whom symptoms persist.

The iliopsoas tendon itself may cause an impingement. Domb [6] described a specific pattern of hip pain associated with a labral injury at the 3 o’clock or direct anterior position with no evidence of FAI, bony abnormality, trauma, or any other known cause of labral injury. Clinical presentation is usually an anterior hip pain and pain exacerbated with active flexion, while some patients also experience a snapping sensation. Iliopsoas tenderness, a positive impingement test, and pain or apprehension with resisted straight leg raise may be apparent at physical examination. MRA may show isolated injury to the labrum at the 3 o’clock position. In some cases, this injury may be associated with an inflamed appearance of the iliopsoas tendon.

While some patients experience incomplete pain relief with intra-articular injection, many found more complete relief after a psoas injection. Arthroscopic release may be performed in patients unresponsive to corticosteroid injection.

**Fig. 2:**
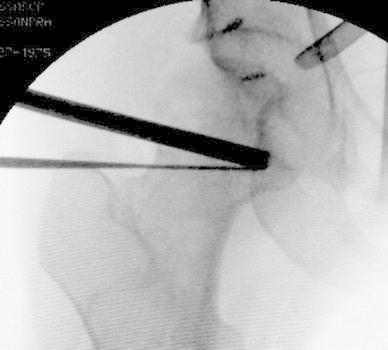
Image intensifier view of transcapsular psoas tendon release

**Fig. 3:**
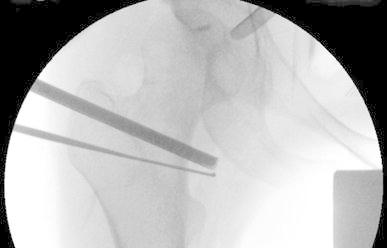
Image intensifier view of psoas tendon release at the level of lesser trochanter

**Fig. 4:**
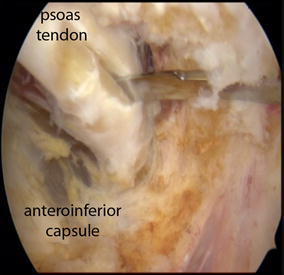
Arthroscopic view of psoas tendon release trough peripheral compartment

Open procedures are reported in the orthopedic literature [7–11] and more recently two distinct arthroscopic techniques [12] have been described for release of the iliopsoas tendon. Tendon release may be performed at the level of the hip joint through an anterior capsulotomy (trans-capsular) [12] (Fig. 2) or at its distal insertion to the lesser trochanter within the iliopsoas bursa (Figs. 3, 4) [12].

The trans-capsular technique allows for release of the tendon via the central or peripheral compartment. The later, described by Ilizaliturri [12], addresses the iliopsoas tendon immediately anterior to the hip capsule in the space between the zona orbicularis and anterior labrum proximal, and anterior to the medial synovial fold. A capsulectomy is performed at this level to gain access to the iliopsoas tendon; synovial tissue from around the tendon may be debrided using a radiofrequency probe or an arthroscopic shaver.

Endoscopic release may also be performed at the lesser trochanter. A spinal needle is guided into position under image intensifier control, aiming for its tip to be located within the iliopsoas insertion. This manoeuvre may be aided by “palpating” the medial aspect of the femur with the tip of the spinal needle as it lands on the lesser trochanter. The needle may then be substituted by an arthroscopic cannula prior to the introduction of a 30° arthroscope. A second working portal is created by triangulation toward the tip of the arthroscope inside the iliopsoas bursa. The image intensifier is used to confirm correct location of the radiofrequency probe prior to iliopsoas tendon release.

Trans-capsular tendon release presents a potential risk of joint instability due to the extensive capsulotomy, especially in patients with hyperlaxity. However, it may be more easily performed via the peripheral compartment. The orthopedic literature reports one paper in which the two techniques were compared, with the author finding no clinical difference in results. Published results of open and arthroscopic techniques are summarized in Table 1 [4, 6–16]. Arthroscopic release appears to be successful and, in general, there are higher success rates and less recurrence with the endoscopic technique compared with open procedures.

**Table 1:** Literature reports on treatments for internal snapping hip syndrome and psoas tendon impingement

First author	References	Technique	Number of hips	Follow-up (months)	Results
Taylor	[7]	Open release (medial approach)	17	17	31 % residual pain, 38 % recurrent snapping, 12.5 % had persistent weakness with hip flexion
Jacobson	[8]	Open Z plasty	20	25	19 reductions of snapping frequency, 14 no snapping, 2 required reoperation, 3 subjective weakness
Dobbs	[9]	Open Z plasty (iliofemoral incision)	11	48	11 had complete pain relief, no patient had detectable loss of hip flexion strength, 1 patient had a recurrence of the snapping, and 2 patients had a transient decrease in sensation over the anterolateral thigh due to injury of the lateral femoral cutaneous nerve
Gruen	[10]	Open Z plasty (ilioinguinal incision)	11	36	58 % complete resolution of their hip pain, 17 % had recurrence, 25 % improved, 5 had postoperative subjective weakness. No complications related to the wound or surgical approach
Hoskins	[11]	Open tendon lengthening (iliofemoral incision)	92	65	12 % recurrence of snapping within 3 months and another 10 % after 3 months, 12 % had surgical incision related complications
Byrd	[4]	Endoscopic release at lesser trochanter	9	20	All patients pain free, no recurrence
Ilizaliturri	[12]	Endoscopic release at lesser trochanter (10) and endoscopic transcapsular (9) (randomized)	19	20	Improvements in WOMAC scores were statistically significant in both groups, and no difference was found in postoperative WOMAC results between groups. No complications were seen
Anderson	[13]	Endoscopic release at lesser trochanter (athletes)	12	9	Preoperative hip scores averaged 41 and 44 points for the competitive and recreational athletes, respectively, at 12 months, 96 and 97 points, and none had recurrence of their snapping or pain
Flanum	[14]	Endoscopic release at the lesser trochanter	6	12	38 points average Harris hip scores increase, no recurrence
Wettstein	[15]	Endoscopic release (preserving iliacus muscle) transcapsular	9	3	No complications, hip flexion strength was restored to normal within 3 months
Ilizaliturri	[16]	Endoscopic release at the lesser trochanter	7	21	All the patients were relieved of their painful snapping symptoms, average WOMAC score improved from 82.5 to 91 points. The only complication seen was loss of flexion strength in all patients
Domb	[6]	Endoscopic tenotomy and either labral debridement or repair	25	21	Mean preoperative HHS, ADL HOS, and Sport HOS scores were 61.64, 73.94, and 51.63, respectively, the mean post-operative scores were 86.06, 88.21, and 72.01, respectively

### Anteroinferior iliac spine impingement

**Fig. 5:**
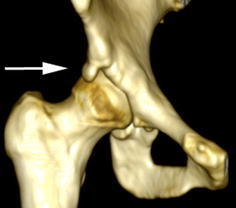
Pre-operative 3-D CT reconstruction image of anteroinferior iliac spine impingement lesion

The anterior inferior iliac spine (AIIS) represents the origin of the direct head of the rectus femoris tendon [17]. Avulsion of the AIIS has been described in the literature after traumatic injury and, if not adequately treated, could result in an anterior impingement against the distal femoral neck (Fig. 5) [17].

Patients usually describe a “grinding sensation” in flexion and rotation of their hip and pain with athletic activity and prolonged hip flexion. Physical examination confirms pain with hip flexion, internal rotation, and adduction and limited hip flexion [18]. Radiographs show a low AIIS, commonly due to prior traumatic avulsion [19].

**Fig. 6:**
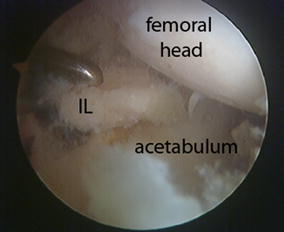
Arthroscopic view at excision of anteroinferior iliac spine impingement lesion (IL)

Open treatment via a direct anterior approach to the AIIS and internal fixation has been described for selected cases [17]. Pan [18] described a 30-year-old man who presented with evidence of impingement between the femoral head-neck junction and an abnormally large anterior inferior iliac spine. Open resection of the hypertrophic anterior inferior iliac spine was performed, which produced full painless restoration of function of the hip (Fig. 6). Larson published two papers describing three and ten cases, respectively, of AIIS or subspine impingement that he managed with arthroscopic decompression [19, 20]. Matsuda [21] has also described arthroscopic management in a case of secondary symptomatic femoroacetabular impingement arising from an inferiorly malunited AIIS avulsion fracture.

Decompression may be performed using arthroscopy of the hip. The AIIS is easily identified by stripping the capsule over the AIIS with a combination of a shaver and burr. However, the authors [20] describe risk of detachment of the rectus femoris from its origin, owing to overzealous resection, which may in turn lead to a potential deficit in hip flexion. Results of open and endoscopic procedures are summarized in Table 2. More studies with larger cohorts and longer follow-ups are necessary to perform a more adequate evaluation of the role of endoscopic techniques in AIIS impingement.

**Table 2:** Literature reports on treatments for anteroinferior iliac spine impingement

First author	References	Procedure	Number of treated hips	Results
Rajasekhar	[17]	Resection of the hypertrophic AIIS (patient 1), 1 fixation of AIIS fragment (patient 2)	2	Patient 1: at 2.5 years follow-up asymptomatic and returned to his normal sporting activity. Patient 2: at 1 year follow-up pain free, able to participate actively in sport
Pan	[18]	Resection of the hypertrophic anterior inferior iliac spine	1	Full painless restoration of function of the hip
Larson	[19]	Arthroscopic decompression	3	Average MHHS: 75.6 points pre op and 93.7 post op; average VAS 6.2 pre op and 1.1 post op
Hetsroni	[20]	Arthroscopic decompression	10	At a mean follow-up time of 14.7 months, the modified Harris hip score improved from 64.18 before surgery to 98.2 points
Matsuda	[21]	Arthroscopic spinoplasty	1	At 18 months follow-up returned to football, a nonarthritic hip score of 98 points, nonrestrictive heterotopic ossification

### Greater trochanteric pain syndrome

Greater trochanteric pain syndrome (GTPS) has an estimated incidence of 1.8 per 1,000 persons [22]. This syndrome includes 3 well-described entities: external coxa saltans, greater trochanteric bursitis, and gluteus medius and/or minimus tears. Although these disorders are often associated, for academic purposes they will be analyzed separately in the flollowing paragraphs [23].

### External snapping of the hip

**Fig. 7:**
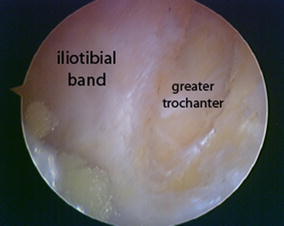
Endoscopic view of iliotibial band Z lengthening

**Fig. 8:**
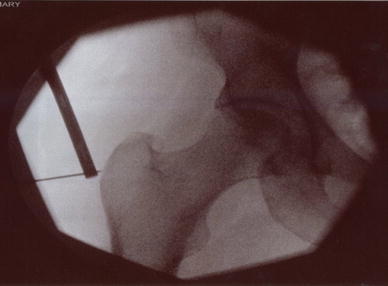
Image intensifier view of endoscopic portal placement for trochanteric procedures

External snapping of the hip is caused by thickening of fibers of the iliotibial band [24], which snap over the greater trochanter with hip flexion and extension and, in more severe cases, with hip rotation. The diagnosis may be confirmed with ultra sound imaging [25]. The patient may be asymptomatic and reproduce the snapping voluntarily. In such cases exercises to stretch the iliotibial band are recommend. In the symptomatic patient, the first line of treatment is physiotherapy. If physiotherapy fails, endoscopic surgical release may be indicated. A recent systematic review [26] found traditional non-operative treatments to be effective in most refractory cases. Open iliotibial band release or lengthening has been the traditional surgical option to treat external snapping hip syndrome [27–31]. Ilizaliturri [32] has recently described a technique for endoscopic iliotibial band release performed under image intensifier control. He describes proximal and distal trochanteric portals through which either a Z- or a diamond-shaped release is performed (Figs. 7, 8). Where indicated, the additional treatment of gluteus medius tear, trochanteric bone prominence or bursitis may be performed as described subsequently. Results of open and endoscopic procedures for lateral hip pathologies are shown in Table 3. According to Ilizariturri’s review [3], endoscopic surgery in the peritrochanteric space seems safe, reproducible, and allows for the successful treatment of snapping hip syndromes.

**Table 3:** Literature reports on treatments for external snapping of the hip, trochanteric bursa inflammation and gluteus medius diseases

First author	References	Technique	Number of hips	Follow-up (months)	Results and complications
Provencher	[27]	Open Z plasty	9	23	All patients had complete resolution of the snapping hip, 1 returned to full unrestricted activities but no residual snapping
White	[28]	Open vertical incision and multiple transverse cuts	16	32.5	14 asymptomatic patients after release (2 hips needed a second release)
Faraj	[29]	Open Z plasty	11	12	Good results, 3 had problems due to scar sensitivity
Fery	[30]	Open cross cut and inverted flap suture	35	84	30 % successful results, 30 % had a recurrence of symptoms and over 60 % continued to experience pain
Govaert	[31]	Open longitudinal release of the iliotibial band combined with excision of the trochanteric bursa	12	23.5	The mean difference between the pre- and postoperative Merle d’Aubigné and Postel scores was 11.7 points; 6 excellent results, 5 good and 1 poor. One screw removal for pain, one surgically drained hematoma
Ilizaliturri	[32]	Endoscopic diamond shape defect	11	25	1 residual nonpainful snapping, 10 excellent results
Wiese	[33]	Endoscopic bursectomies and in 4 coxa saltans suture of the iliotibial tract to the greater trochanter	37	25	32.5 points was the average Japanese orthopedic association score improvement; VAS improved from 7.2 to 3.8 points. Four patients developed hematoma
Baker	[34]	Endoscopic bursectomy	30	26.1	VAS improved from 7.2 to 3.1, mean Harris hip scores improved from 51 to 77 points, one seroma and one subsequent open bursectomy
Fox	[35]	Endoscopic bursectomy	27	60 (only for 22 patients)	24 good or excellent results without complications, 2 recurrences, 1 unsatisfied
Bradley	[36]	Endoscopic bursectomy	2	7	Immediate symptomatic improvement, returned to competitive basketball with occasional aching in his right hip
Farr	[37]	Endoscopic bursectomy and concomitant iliotibial band release under local anesthesia	2	41	All excellent results, no recurrence
Kandemir	[38]	Endoscopic excision of gluteus medius/minimus calcifications	1	3	Symptom-free without limitation of any activity, normal abduction strength
Voos	[39]	Endoscopic repair of gluteus medius/minimus tears	10	25	10 complete resolution of pain; no adverse complications. Seven of 10 patients said their hip was normal, and 3 said their hip was nearly normal
Davies	[40]	Open suture of torn abductors with soft-tissue anchors in the greater trochanter	16	12	4 re-ruptures, 1 deep infection. In the remaining 11 patients there were statistically significant improvements in VAS and Oxford hip score

### Trochanteric bursa inflammation

Inflammation of the trochanteric bursa is the most common cause of pain over the lateral aspect of the hip [23]. Symptoms may be aggravated by pressure over the area, weight bearing, and resisted external rotation in supine (with hip flexed to 90°) and prone (with hip extended) positions.

The improved accuracy of MRI has allowed for more precise diagnosis of bursitis [41]. MRI is also helpful to rule out gluteus medius tears that may present with similar symptoms [41].

The first line of treatment is conservative, with appropriate anti-inflammatory therapy and lifestyle modification [42]. Corticosteroid injections have been commonly utilized in the presence of failed conservative treatment. Bursitis often responds favorably to conservative management [43]. When the bursitis is refractory to conservative treatment and to corticosteroid injection, surgical bursectomy should be considered [43].

**Fig. 9:**
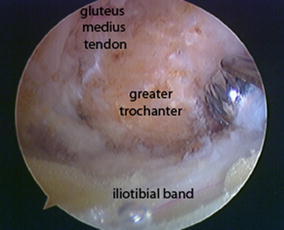
Endoscopic view of greater trochanter prominence excision after trochanteric bursectomy

Endoscopic bursectomy [34–37] may be performed in either the lateral decubitus or supine positions [42]. In the lateral position, bursectomy can be performed through a proximal portal 2-cm posterior to the tip of the trochanter and a distal portal at the postero-inferior margin of the bursa. A 4-mm, 30° arthroscope can be used to examine the bursa through the posterior distal portal. The leg is abducted to relax the soft tissues and maximize distention in the bursa. Debridement can be performed with a 4-mm full-radius resector placed in the proximal portal. When the bursectomy is complete, the arthroscope and shaver are switched for a final check. If there is an associated prominence of the greater trochanter, the bone can be shaved with a 5.5-mm burr (Fig. 9). In our experience the lateral position technique lends itself more readily to bursectomy with portals made through the tensor fascia lata, and a Z-shaped fasciotomy performed if working space is inadequate. Strauss [42] described an endoscopic technique in the supine position, which preserves the fascia lata if a fasciotomy is not required. This technique has gained recent popularity and is performed through the anterior portal (1 cm lateral to the anterior superior iliac spine and in the interval between the tensor fascia lata and the sartorius) and the distal peritrochanteric space portal (DPSP: midway between the tip of the greater trochanter and vastus tubercle along the posterior one-third of the greater trochanter midline). The leg is positioned in full extension and approximately 15° of abduction. The shaver may then be placed within the peritrochanteric space through the DPSP to begin bursectomy. Evaluation of the iliotibial band, vastus lateralis insertion, gluteus minimus tendon and muscle and the insertion of the gluteus medius tendon at the greater trochanter may be performed. If a torn tendon is identified it may be repaired with use of suture anchors. Release of the iliotibial band is performed only if necessary for either external coxa saltans or abnormal contact across the greater trochanter. Results of endoscopic bursectomies are analyzed in Table 3 and showed a good efficacy and a low rate of complications.

### Gluteus medius diseases

When GTPS is caused by a gluteus medius tendinopathy, the majority of patients describe lateral-sided hip pain of insidious onset that is aggravated with weight bearing and resisted hip abduction [23]. Patients can often point to an exact point of pain that often occurs some centimeters proximal to the tip of greater trochanter. Good quality MRI is necessary to discriminate gluteal tears and trochanteric bursitis [41]. Once again, physiotherapy is the first line of treatment. When this fails, surgical treatment via various open [40] and more recently described endoscopic techniques is an option. Two endoscopic repair techniques of the gluteus tendon are described in the literature [39–44].

Voos et al. [39], describes a technique to repair gluteus tendon tears via the peritrochanteric compartment utilizing an anterior portal and a distal posterior portal.

**Fig. 10:**
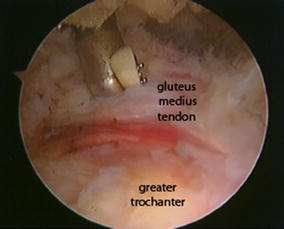
Endoscopic view of gluteus medius tendinosis

Here the peritrochanteric space is typically distended with 50–70 mm Hg of pressurization. The attachment site of the tendon at the greater trochanter (Fig. 10) is prepared with a full-radius shaver similar to preparation of the footprint for rotator cuff tears. Suture anchors can be placed into the footprint of the abductor tendons, the sutures are passed sequentially through the edges of the prepared gluteus medius tendon with a suture-passing device and tied. Voos [39] reported complete resolution of symptoms in all ten patients in his series using this technique.

Domb [44] described another endoscopic technique of approaching the tendon through a longitudinal split in line with its fibers, in order to visualize and treat intra-substance tears without affecting the integrity and strength of the tendon itself. He describes the use of posterolateral and distal peritrochanteric portals. This technique results in a side-to-side repair of the longitudinal tendon split while firmly approximating the tendon to the footprint on the lateral facet.

As shown in Table 3, both techniques showed a good efficacy. According to our view, Domb’s procedure [44] appears more challenging for the surgeon and aims to better preserve the tendon, which may be helpful in younger and more active patients. Studies with a larger cohort are necessary to give an evidence-based conclusion for the efficacy of these endoscopic techniques.

### Piriformis syndrome

Piriformis syndrome results from sciatic nerve entrapment by the piriformis muscle [45]. It manifests as buttock pain and radicular-like pain associated with hip flexion movements with combined internal or external rotation. Piriformis syndrome often occurs after blunt trauma to the buttock with resultant hematoma formation and subsequent scarring between the sciatic nerve and external rotators [45]. Clinical tests such as Lasègue, Pace and Friberg sign are commonly positive and useful in the diagnosis of sciatic nerve entrapment.

Nerve conduction studies and MRI may confirm the clinical suspicion and exclude other causes of entrapment [46, 47].

Open surgical techniques have been shown to reduce pain associated with entrapment of the sciatic nerve caused by fibrous scar bands, vascular structures, or muscular anatomy [47–49].

Endoscopic treatment of piriformis syndrome under local anesthesia has been described [50–52]. As previously described, the portals for the peritrochanteric space are commonly utilized. A posterolateral auxiliary portal (3 cm posterior and 3 cm superior to the greater trochanter) may be used in the presence of variation of sciatic nerve entrapment (atypical fibrous bands, hamstring tendons or fibrous scar bands extending from the trochanteric bursa to the sciatic nerve). Open and endoscopic results are compared in Table 4. Martin et al. [50] advocate endoscopic sciatic nerve decompression be performed only by hip arthroscopy experts and attribute their results to careful patient selection and surgical experience. Further studies are required to extend the indication of this procedure to less expert surgeons.

**Table 4:** Literature reports on treatments for piriformis syndrome

First author	References	Procedure	Number of hips	Follow-up	Results and complications
Benson	[47]	Open excision of post-traumatic adhesions	16	38 months	Eleven excellent and four good results, one seroma and one infected hematoma
Fishman	[48]	Open nerve decompression	43	16 months	68.8 % patients showed 50 % or greater improvement; mean improvement was 68 %
Filler	[49]	Open nerve decompression (3 cm incision, transgluteal approach)	62	Unstated	Excellent outcome in 58.5 %, good outcome in 22.6 %, limited benefit in 13.2 %, no benefit in 3.8 %, and worsened symptoms in 1.9 %
Martin	[50]	Endoscopic nerve decompression, piriformis tendon release or by hamstring tendon scarring	35	1 year	The mean postoperative MHHS increased to 78.0 and VAS score decreased to 2.4. The mean postoperative MHHS increased to 78.0 and VAS score decreased to 2.4
Dezawa	[51]	Endoscopic release of the piriformis muscle under local anesthesia	8	Unstated	Good results in all patients
Hwang	[52]	Endoscopic incision, drainage of benign cystic lesion on the sciatic nerve and release of the piriformis tendon	1	20 months	Good result, no recurrence

### Hamstring avulsion and tendinopathy

**Fig. 11:**
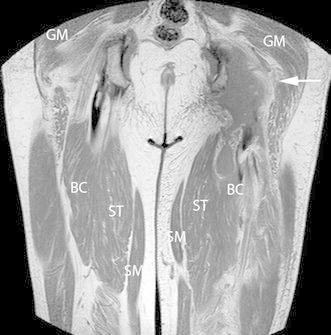
MRI of left hamstring avulsion (*GM* gluteus maximus, *BC* biceps femori, *ST* semitendinosus, *SM* semimembranosus)

Complete hamstring tendon rupture can occur during sports or slipping on a slick surface while hamstring tendinopathy is likely caused by a mixture of hereditary, structural, professional, and lifestyle factors [53]. The peak age for injury is the early to mid forties but it can occur also in the age range of 18–20 years [54]. In those cases the degree of trauma is typically more severe due to the external overload placed on the normal tendon. The majority of patients report being forced into a position of extreme hip flexion with the knee near or at full extension. A minority of patients describes the mechanism of injury to resemble “doing the splits”. Patients often describe feeling a “pop” or tearing sensation in the hamstring/gluteal region. Patients usually find it difficult to extend the knee, presumably due to muscle spasm or irritation of the sciatic nerve. The presence of considerable swelling and ecchymosis along the posterior thigh may be seen in the acute setting and the patient may describe sciatica symptoms. Isometric testing of the hamstrings with knee flexed to 70° may reveal significant deformity of the affected hamstring muscle belly owing to its distal retraction [54]. Positive patient history and clinical findings at physical examination should prompt further assessment with an MRI scan even when a complete avulsion is suspected clinically, as this would allow for precise delineation of the injury (Fig. 11) [55].

Surgical repair is indicated for patients in their youth, in athletes and those who have sustained acute complete tear of all three tendons with more than 2 cm retraction. Chronic tendinopathy on the other hand is commonly treated conservatively [56]. Surgical debridement of scar may be indicated in cases where conservative treatment has failed. Open surgical techniques [56–59] for hamstring avulsions and tendinopathy are described in the literature, and recently Guanche described an endoscopic technique [60]. Endoscopic treatment is performed in the prone position through three portals. The first two portals are created 2 cm medial and lateral to the ischial tuberosity. The lateral portal is first used for a 30° arthroscope. The tip and medial aspect of the ischium are delineated, then the lateral aspect is exposed with the use of radiofrequency. With the lateral aspect identified, the dissection continues anteriorly and laterally towards the sciatic nerve. A careful release of soft tissue is performed to cautiously mobilize the nerve and avoid inadvertent injury. The tendinous origin is then inspected for any obvious tearing. A more inferior portal may then be created approximately 4 cm distal to the tip of the ischium and equidistant from the medial and lateral portals. This portal is employed either for insertion of suture anchors or suture management.

In Table 5 the available endoscopic results have been compared with published results obtained through open procedures. Although endoscopic treatment appears a good option in expert hands, at present there is just a single case series published in the literature. Therefore, more evidence is required if surgeons are to transfer more widely to endoscopic techniques.

**Table 5:** Literature reports on treatments for hamstring avulsion and tendinopathy

First author	References	Number of hips	Treated lesions	Results
Allay	[54]	25	18 patients acute hamstring avulsion, 7 patients chronic tears	92 % minimal or no pain, 96 % estimated their functional recovery to be greater than 75 % of the uninvolved limb, and 88 % felt their strength was greater than 75 % of the uninvolved limb. Subjects who were isokinetically tested more than 1 year after surgery averaged 98 % strength compared to the uninvolved limb
Folsom	[57]	26 (athletes)	21 patients acute hamstring avulsion, 5 patients chronic tears	76 % percent of their patients returned to sports.Overall, 96 % of athletes reported good leg control, and 80 % of athletes were pain free
Sarimo	[58]	41	Complete avulsions	At an average of 37 months 90 excellent results, 10 were good, 5 were moderate, and 7 were poor. Six of 7 poor results were in patients treated >3 months post injury
Wood	[59]	71	47 complete retracted tears, 16 complete and minimally retracted tears, 7 incomplete tears, 1 severe muscle-tendon rupture, 1 ischial tuberosity avulsion	At 2 years postoperatively 84 % strength. 80 % of patients returned to their previous sporting activities. There were statistically inferior hamstring strength and endurance results in patients with chronic retracted tears when compared to the remaining patients
Guanche	[61]	15	Acute hamstring avulsion (3 patients), partial hamstring avulsion (9 patients), ischial bursitis (3 patients)	One patient (with preoperative refractory ischial bursitis) had recurrent ischial pain, 2 patients complained of numbness over the posterior thigh with resolution of their symptoms by 6 weeks postoperatively

## Conclusions

Open surgery has been utilized for many years to address various extra-capsular pathologies of the hip. Endoscopic techniques have since evolved to treat internal snapping hip syndrome, iliopsoas impingement, AIIS impingement, external snapping hip syndrome, trochanteric bursitis, gluteus medius tears, piriformis syndrome and proximal hamstring diseases. Although the current evidence for these endoscopic techniques is limited to single case series, published results are encouraging. Excellent results are not reported in all patients with either open, arthroscopic or endoscopic procedures. There is consensus about the importance of accurate patient selection to achieve the best results. Further randomized studies would be necessary to justify the choice of arthroscopic or endoscopic procedures, perhaps based on comparability of success rates, complication rates and attendant advantages of day-case surgery.
